# The utility of the Diabetes Anxiety Depression Scale in Type 2 diabetes mellitus: The Fremantle Diabetes Study Phase II

**DOI:** 10.1371/journal.pone.0194417

**Published:** 2018-03-15

**Authors:** Wendy A. Davis, David G. Bruce, Milan Dragovic, Timothy M. E. Davis, Sergio E. Starkstein

**Affiliations:** 1 Medical School, University of Western Australia, Perth, Western Australia, Australia; 2 Clinical Research Centre, North Metropolitan Health Service Mental Health, Perth, Australia; 3 School of Psychiatry, University of Western Australia, Perth, Western Australia, Australia; Nagoya University, JAPAN

## Abstract

**Background:**

Previous research using latent class analysis (LCA) identified classes of people with type 2 diabetes and specific profiles of depression and anxiety. Since LCA-derived anxious depression strongly predicts cardiovascular outcomes and mortality but cannot be applied to individuals, we developed a validated combined depression-anxiety metric, the Diabetes Anxiety Depression Scale (DADS), for potential clinical application in people with type 2 diabetes.

**Methods:**

1,337 participants with type 2 diabetes from the observational community-based Fremantle Diabetes Study Phase II completed the Patient Health Questionnaire 9-item version (PHQ-9) to assess symptoms of depression, and the Generalised Anxiety Disorder Scale (GADS) to assess symptoms of anxiety. A single score was calculated by adding all the PHQ-9 items and the four GADS items used for the LCA. Cut-off scores were calculated with Receiver Operating Characteristic (ROC) area under the curve (AUC).

**Results:**

The optimum cut-off scores in terms of sensitivity, specificity, positive and negative predictive value were 18 points for major anxious depression and 8 points for minor anxious depression. A score of 8–17 was associated with a significantly increased incidence of coronary heart disease, whereas a score 18–39 was associated with an increase in both coronary heart disease and cardiovascular mortality.

**Conclusions:**

The DADS has strong psychometric validity in the identification of mixed depression-anxiety in type 2 diabetes, and may contribute to cardiovascular risk prediction.

## Introduction

Depression is common in people with type 2 diabetes but there are still questions regarding its phenomenology and optimal screening methods [1,2]. We recently used latent class analysis (LCA) to demonstrate the presence of specific classes of combined depression and anxiety, which we termed major and minor anxious depression, and subclinical anxiety, at baseline in a prospectively studied cohort of 1,337 community-based people with type 2 diabetes [3]. LCA is a statistically strong technique for symptom classification. It assumes that a population of individuals is a mixture of distinct but internally homogeneous subgroups and it identifies classes of people that do not overlap in terms of their pattern of symptoms. This simple categorization provided by LCA improved the prediction of subsequent adverse outcomes including cardiovascular events and associated mortality after adjusting for conventional risk factors [4].

**Fig 1:**
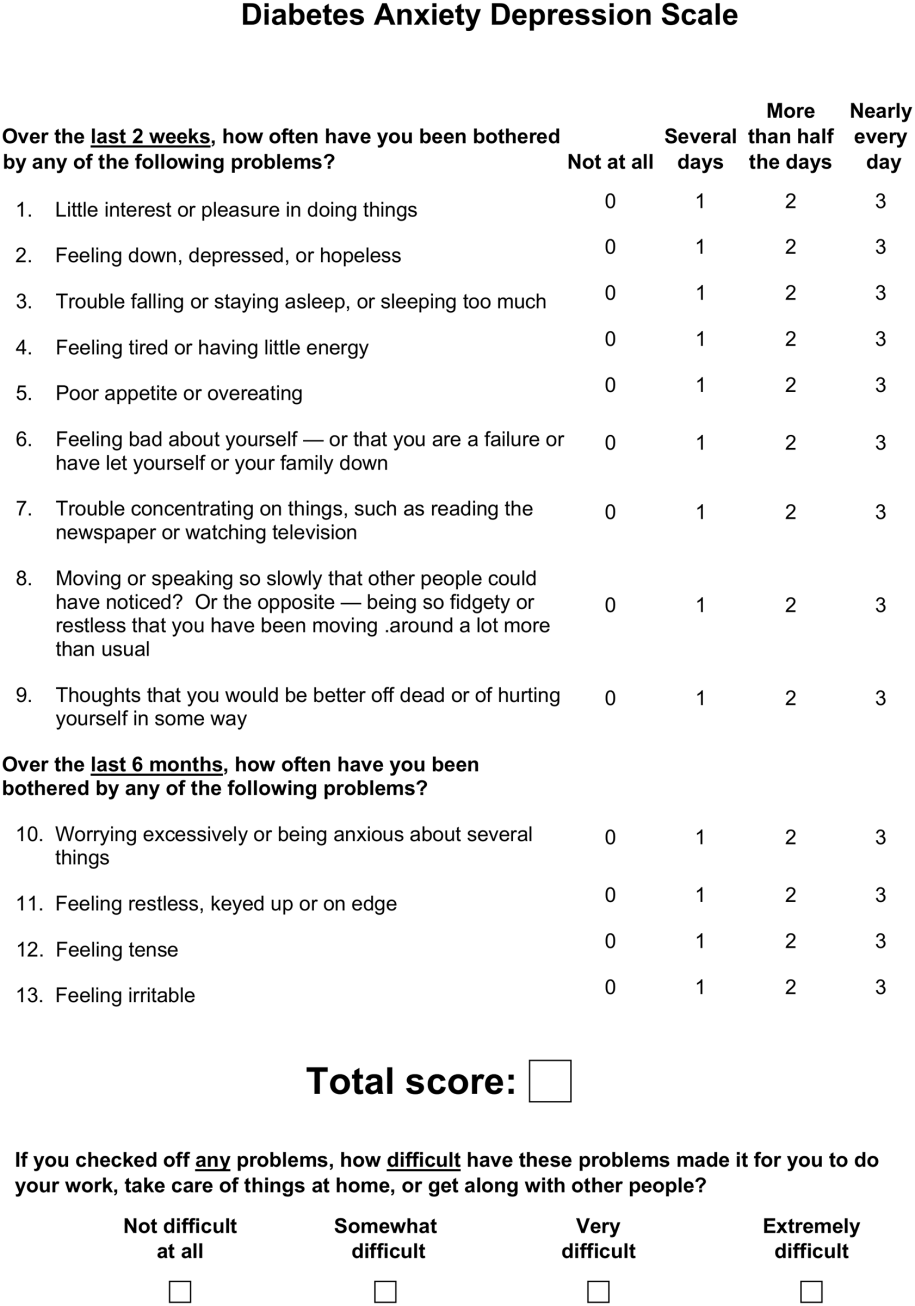
The Diabetes Anxiety Depression Scale.

Since LCA can only be applied to groups of people, the next step in clinical translation is to identify categories of depression and anxiety types at the level of the individual. The aim of the present study was, therefore, to derive a simple scoring system from a single instrument (the Diabetes Anxiety Depression Scale (DADS); see Fig 1) that combines the 9-item Patient Health Questionnaire (PHQ-9; an instrument commonly used to screen for depression in diabetes) [5] and four items of the Generalised Anxiety Disorder Scale (GADS; an instrument validated for screening anxiety in type 2 diabetes) [3].

## Methods

### Study sample

Participant characteristics have been detailed in the previous report of LCA-derived classes [3]. Briefly, the sample comprised 1,337 of the 1,551 participants (86%) with clinically defined type 2 diabetes recruited into the Fremantle Diabetes Study Phase II (FDS2) between 2008 and 2011, who had complete PHQ-9 and GADS data. The FDS2 is a longitudinal study of known diabetes conducted in a postcode-defined geographic area surrounding the port city of Fremantle in the state of Western Australia (WA).

### Ethics statement

The Human Research Ethics Committee of the Southern Metropolitan Area Health Service approved the study, and all participants gave written informed consent.

### Depression and anxiety assessment

All patients completed the PHQ-9 which consists of nine items corresponding to the nine DSM-5 criteria for major depression. Each item is rated as 0 (symptom is not present), 1 (symptom present for several days), 2 (symptom present for more than half the days), or 3 (symptom present nearly every day). The GADS consists of two items corresponding to the A and B DSM-5 mandatory criteria for generalised anxiety disorder, the 6 items rating clinical symptoms, and a final item rating the DSM-5 mandatory criterion for distress, with a rating scheme the same as that for the PHQ-9. The psychometric attributes of the GADS have been described [3]. For the present study, the total PHQ-9 score was added to the four GADS items scores (worrying, feeling irritable, muscle tension, and restlessness) used in the LCA analysis which defined four categories of anxious depression (none, subclinical anxiety, minor, and major anxious depression) [3].

### Clinical and outcomes assessment

Full details of the clinical and outcomes assessment have been reported [4]. Briefly, each participant completed comprehensive questionnaires, a physical examination was conducted, and fasting biochemical tests were performed in a single nationally accredited laboratory. All hospital admissions and deaths in the state of WA are recorded in the WA Data Linkage System and these were linked to the FDS2 cohort from 1970 to end-June 2013. Causes of death, based on information provided on the death certificate or by the coroner’s determination, were reviewed independently by two study physicians (DGB and TMED) and classified under the system used in the UK Prospective Diabetes Study [6]. Cardiovascular and all-cause mortality and incident hospitalisation for coronary heart disease (CHD) from FDS2 study entry to end-June 2013 were determined. Incident CHD was defined by identifying the first hospitalisation for/with CHD or death from cardiac or sudden causes, if there was no prior hospitalisation.

### Statistical analysis

As well as the individual DADS score, participants were categorized by LCA-derived anxious depression class as described previously [3]. The area under the Receiver Operating Characteristic (ROC) curve (AUC) was used to define the optimum cut-offs at the maximum value of the Youden Index (Sensitivity + Specificity– 1) for i) major anxious depression, and ii) no anxious depression from the complete dataset used for the LCA (N = 1,337). Having defined these classes at the extremes of the total score distribution (n = 124 for major anxious depression (total score ≥18) and n = 446 for no anxious depression (total score ≤2)), a third ROC AUC analysis was performed contrasting subclinical anxiety and minor anxious depression in the remaining 767 subjects who had scores in the range 3–17. Sensitivity, specificity, positive predictive value (PPV), and negative predictive value (NPV) were derived for all for the optimum total score ranges compared with the LCA-derived anxious depression classes as gold standard.

## Results

The 1,337 participants in the present study had a mean±SD age of 65.8±11.1 years, 54% were males, their median [inter-quartile range] diabetes duration was 8.8 [2.8–15.6] years, and their median HbA_1c_ was 51 [44–60] mmol/mol (6.8 [6.2–7.6]%). The remaining 214 patients with missing data were less likely to be male (43%, *P* = 0.005) and had worse glycaemic control (HbA_1c_ 53 [46–69] mmol/mol (7.0 [6.4–8.5]%), *P* = 0.001), but there were no significant between-group differences in age (64.9±14.4 years, *P* = 0.41) or diabetes duration (10.0 [3.0–17.8] years, *P* = 0.12).

The DADS has high internal consistency (Cronbach’s alpha = 0.91). The total DADS score categorized by LCA-derived anxious depression group is shown in Table 1. The ranges of DADS overlapped between groups, especially between those with subclinical anxiety and minor anxious depression. A first ROC analysis was therefore undertaken on all 1,337 individuals with LCA-derived major anxious depression (n = 104) as the outcome. A cut-off of 17.5 had the highest sensitivity, specificity, PPV and NPV (see Table 2) for identifying individuals with and without major anxious depression, but since the total score is in whole numbers this was rounded up to 18 thus identifying 124 individuals with DADS-defined major anxious depression.

**Table 1:** Descriptive statistics for total score on the Diabetes Anxiety Depression Scale (DADS) by latent class analysis (LCA)-derived anxious depression class.

		DADS	total	score
	N	Mean ± SD	Median [IQR]	Range
**LCA-derived anxious depression classes:**				
No anxious depression	439	0.8 ± 0.9	0 [0–1]	0–4
Subclinical anxiety	501	5.2 ± 2.1	5 [4–6]	2–12
Minor anxious depression	293	12.3 ± 3.2	12 [10–15]	5–20
Major anxious depression	104	25.6 ± 5.3	24 [22–29]	18–39

**Table 2:** Differentiation of i) no anxious depression from the other classes, ii) major anxious depression from the other classes, and iii) subclinical anxiety from minor anxious depression using total Diabetes Anxiety Depression Scale score.

	No anxious depression	Major anxious depression	Subclinical anxiety	Minor anxious depression
**N**	1,337	1,337	767	767
**AUC (95% CI)**	0.008 (0.005–0.011)	1.000 (0.999–1.000)		0.969 (0.959–0.979)
**Optimum cut-off**	2.5	17.5		7.5
**Range used**	0–2	18–39	3–7	8–17
**Sensitivity**	0.945	1.000	0.849	0.945
**Specificity**	0.965	0.984	0.869	0.856
**Positive predictive value**	0.930	0.839	0.911	0.784
**Negative predictive value**	0.973	1.000	0.784	0.966

AUC: area under the receiver operating characteristic curve; CI: confidence interval

A second ROC analysis was performed on the same 1,337 individuals but this time with LCA-derived no anxious depression (n = 439) as the outcome. A cut-off of 2.5 had the highest sensitivity, specificity, PPV and NPV (Table 2) for identifying individuals with and without LCA-derived no anxious depression but this was rounded down to 2, a score which classified 446 individuals with DADS-defined no anxious depression. Every participant with LCA-derived major anxious depression was correctly classified (see Table 2). Moreover, only 1.5% of the sample (n = 20) was incorrectly classified as having major anxious depression when they had LCA-derived minor anxious depression. Of the 439 with LCA-derived no anxious depression, 415 (94.5%) were correctly identified with a DADS score ≤2 (Table 2), with the remaining 24 being grouped within DADS-defined subclinical anxiety at the next step.

The third ROC AUC analysis was performed in the remaining 767 subjects who had scores in the range 3–17 with LCA-derived minor anxious depression (n = 293) as the outcome. A cut-off score of 7.5, rounded up to 8, had the highest sensitivity, specificity, PPV and NPV (see Table 2) in differentiating between subclinical anxiety and minor anxious depression. There was good agreement between the LCA- and DADS-defined classes of major anxious depression, minor anxious depression, subclinical anxiety, and no anxious depression (kappa = 0.90, 0.78, 0.77 and 0.91, respectively).

The identification of LCA-derived anxious depression classes by total score in the whole sample, combining the results of the three ROC analyses, is shown in S1 Table. A total of 258 (88.1%) individuals with LCA-derived minor depression were classified correctly while 15 (5.1%) were classified with subclinical anxiety and 20 (6.8%) with major anxious depression. Likewise, 399 (79.6%) with LCA-derived subclinical anxiety were correctly classified, with 31 (6.2%) being classified with no anxious depression and 71 (14.2%) with minor anxious depression. The proportions of individuals in the four different classes based on LCA, as well as a classification into major depression, minor depression, generalised anxiety disorder and no affective or anxiety disorder based on DSM-5 criteria, are shown in S2 Table. The distribution of individuals based on LCA-derived classes or on the score ranges was almost identical.

During 4,953 person-years (3.7±1.0 years) of follow-up until death or June 30, 2013, 110 (8.2%) participants died, 31 (2.3%) from cardiovascular disease (CVD). During 4,576 person-years (3.4±1.2 years) of follow-up, 199 (14.9%) participants had incident CHD events (first hospitalization for/with CHD or CVD death) [4]. Adjusted hazard ratios (95% confidence intervals) for all-cause mortality, cardiovascular mortality and incident coronary heart disease for both the LCA-derived classes and for groups based on ROC analysis are shown in Table 3. This has previously been reported for the LCA-derived classes [4] but was repeated for the DADS-defined classes for comparison. For either classification there was a ‘dose- response’ relationship with more severe LCA-derived anxious-depression syndromes and higher scores on the DADS being associated with worse outcomes. More specifically, the range of scores 8 to 17 was associated with a significantly increased incidence of coronary heart disease, whereas a score 18 to 39 was associated with an increase in both coronary heart disease and cardiovascular mortality.

**Table 3:** Adjusted hazard ratios (95% CI) for all-cause mortality, cardiovascular mortality and incident coronary heart disease predicted by anxious depression classes derived using latent class analysis (LCA) [4], and by cut-off scores on the Diabetes Anxiety Depression Scale (DADS) for different levels of anxious depression. In each model, the psychiatric categories were added to the most parsimonious Cox proportional hazards model of independently non-psychiatric predictors of the clinical outcome described previously [4].

Psychiatric category	All-cause mortality	Cardiovascular mortality	Incident coronary heart disease
**LCA-derived anxious depression classes:**			
No anxious depression	1.00 (reference)	1.00 (reference)	1.00 (reference)
Subclinical anxiety	1.08 (0.67–1.73)	1.71 (0.70–4.14)	1.40 (0.97–2.03)
Minor anxious depression	1.12 (0.66–1.90)	1.44 (0.46–4.48)	1.70 (1.15–2.50)^b^
Major anxious depression	1.53 (0.74–3.17)	4.28 (1.34–13.68)^a^	1.90 (1.11–3.25)^a^
**DADS scores:**			
0–2	1.00 (reference)	1.00 (reference)	1.00 (reference)
3–7	1.25 (0.77–2.04)	1.39 (0.57–3.40)	1.30 (0.89–1.92)
8–17	1.24 (0.74–2.09)	1.50 (0.53–4.27)	1.90 (1.30–2.77)^b^
18–39	1.56 (0.78–3.16)	3.38 (1.07–10.66)^a^	1.87 (1.11–3.15)^a^

^a^*P*<0.05,

^b^*P*<0.01.

## Discussion

We examined whether membership of LCA-derived classes of anxious-depression in type 2 diabetes could be correctly identified in an individual using the DADS, an instrument combining scores from the PHQ-9 and the GADS. There were two important findings. First, the DADS score, produced by adding the total score of the PHQ-9 with four items from the GADS, was a reliable surrogate for LCA-derived categories of anxious depression. Using ROC analysis, a score above 17 accurately identified major anxious depression while a cut-off score of 8 accurately identified minor anxious depression. Second, scores ≥8 on the DADS were associated with an increased incidence of coronary heart disease whereas scores ≥18 were also associated with increased cardiovascular mortality.

Several existing scales such as the Hospital Anxiety and Depression Scale (HADS) and the Depression Anxiety and Stress Scale (DASS) have been used to screen for depression and anxiety in type 2 diabetes. We selected the PHQ-9 given that this instrument has been extensively validated in primary care and diabetes [2]. The PHQ-9 is used to assess current depressive symptoms and severity, and is the only instrument to screen for the presence of major depressive disorders based on DSM-IV/5 criteria. We designed the GADS to map directly into the nine DSM-IV/5 criteria for Generalised Anxiety Disorder. The GADS was structured using the PHQ-9 as a template, thus allowing for a psychometrically robust amalgamation. We demonstrated that the GADS has high diagnostic concordance with the Structured Clinical Interview for DSM-IV/5 (kappa = 0.88) and high test-retest reliability (intraclass correlation coefficient, ICC = 0.88) [3]. However, to our knowledge, the GADS has not yet been validated in another sample.

The limitations of our approach should be acknowledged. We designed the GADS for an epidemiological study before the GAD-7 [7] was published. Nevertheless, we have demonstrated adequate reliability and validity for the GADS [3]. The second limitation is that we did not assess the category of ‘diabetes distress’ [8] and it is possible that there is some overlap with the LCA-derived syndromes of anxious-depression. Nevertheless, we demonstrated that the scores derived from the DADS have strong clinical relevance. Finally, the current study did not have a validation dataset and did not test for test-retest reliability.

Depression has been identified as a frequent co-morbid condition in Type 2 diabetes, which is itself associated with worse outcomes including increased mortality [9,10]. The frequency and clinical correlates of generalised anxiety disorder in Type 2 diabetes have been less well studied than depression, but there have been several reports of an association between anxiety and increased disability [9,11,12,13]. In the present study, a DADS cut-off score of 18 correctly identified all participants with LCA-derived major anxious depression and was significantly associated with incident coronary heart disease and cardiovascular mortality, while only 6.8% with LCA-derived minor anxious depression were misclassified as having major anxious depression. Similarly, a DADS score greater than seven identified the vast majority of participants with minor depression, with 14.2% of patients with LCA-derived subclinical anxiety misclassified as having minor anxious depression. Overall, 88.0% of participants in the cohort were correctly classified, 3.4% were under-diagnosed and 8.6% over-diagnosed.

## Conclusions

We believe the DADS is a potentially useful instrument in screening for anxious depression in people with type 2 diabetes in clinical practice. Future studies may demonstrate the usefulness of this instrument to monitor response to psychiatric treatment, and for inclusion in assessment of cardiovascular risk.

## Supporting information

S1 Table Quality of identification of latent class analysis (LCA)-derived anxious depression classes by total Diabetes Anxiety Depression Scale (DADS) score (n (%)). (DOCX)

S2 Table Proportions with depression and/or generalised anxiety disorder (GAD) by latent class analysis (LCA)-derived and total Diabetes Anxiety Depression Scale (DADS) score-defined anxious depression classes (N = 1,337). (DOCX)

S1 File Minimal anonymized baseline data set necessary to replicate the main study findings. (XLSX)
