# Supplementary material for: The utility of the Diabetes Anxiety Depression Scale in Type 2 diabetes mellitus: The Fremantle Diabetes Study Phase II
Source: PLoS One. 2018 Mar 15;13(3):e0194417. doi: 10.1371/journal.pone.0194417 (PMC5854400; doi:10.1371/journal.pone.0194417)
Supplement: S1 Table — (DOCX) [file pone.0194417.s001.docx]

**S1 Table.** **Quality of identification of latent class analysis (LCA)-derived anxious depression classes by total Diabetes Anxiety Depression Scale (DADS) score (n (%)).**

|  | **DADS scores:** | | | | |
| --- | --- | --- | --- | --- | --- |
| **LCA-derived anxious depression classes:** | 0-2 | 3-7 | 8-17 | 18-39 | **Total** |
| No anxious depression | 415 (31.0) | 24 (1.8) | 0 (0) | 0 (0) | 439 (32.8) |
| Subclinical anxiety | 31 (2.3) | 399 (29.8) | 71 (5.3) | 0 (0) | 501 (37.5) |
| Minor anxious depression | 0 (0) | 15 (1.1) | 258 (19.3) | 20 (1.5) | 293 (21.9) |
| Major anxious depression | 0 (0) | 0 (0) | 0 (0) | 104 (7.8) | 104 (7.8) |
| **Total** | 446 (33.4) | 438 (32.8) | 329 (24.6) | 124 (9.3) | 1,337 (100.0) |
