# Supplementary material for: The utility of the Diabetes Anxiety Depression Scale in Type 2 diabetes mellitus: The Fremantle Diabetes Study Phase II
Source: PLoS One. 2018 Mar 15;13(3):e0194417. doi: 10.1371/journal.pone.0194417 (PMC5854400; doi:10.1371/journal.pone.0194417)
Supplement: S2 Table — (DOCX) [file pone.0194417.s002.docx]

**S2 Table.** **Proportions with depression and/or generalised anxiety disorder (GAD) by latent class analysis (LCA)-derived and total Diabetes Anxiety Depression Scale (DADS) score-defined anxious depression classes (N=1,337).**

|  | **DSM-5 diagnoses** | | | | | |
| --- | --- | --- | --- | --- | --- | --- |
|  | **No GAD/ no depression** | **No GAD, minor depression** | **No GAD, major depression** | **GAD, no depression** | **GAD, minor depression** | **GAD, major depression** |
| **LCA-derived anxious depression classes:** |  |  |  |  |  |  |
| No anxious depression | 32.8 | 0 | 0 | 0 | 0 | 0 |
| Subclinical anxiety | 36.8 | 0.7 | 0 | 0 | 0 | 0 |
| Minor anxious depression | 15.7 | 4.5 | 0.9 | 0.7 | 0.1 | 0 |
| Major anxious depression | 0.8 | 0.4 | 2.5 | 0.8 | 0.4 | 2.7 |
| **DADS scores:** |  |  |  |  |  |  |
| 0-2 | 33.4 | 0 | 0 | 0 | 0 | 0 |
| 3-7 | 32.1 | 0.7 | 0 | 0 | 0 | 0 |
| 8-17 | 19.3 | 4.2 | 0.4 | 0.6 | 0.1 | 0 |
| 18-39 | 1.4 | 0.7 | 3.0 | 0.9 | 0.5 | 2.7 |
